# Complex perioperative course in a patient with severe rheumatic mitral stenosis: left ventricular rupture, severe left ventricular dysfunction, and mechanical valve dysfunction: a case report

**DOI:** 10.1093/ehjcr/ytag453

**Published:** 2026-06-16

**Authors:** Yingjie Zhang, Jian Wu, Yaxiong Li, Jianye Yang, Maodong Yang

**Affiliations:** Department of Cardiovascular Surgery, Yan’an Affiliated Hospital of Kunming Medical University, No. 245 East Renmin Road, Kunming 650051, China; Institute of Cardiovascular Surgery of Yunnan Province, No. 245 East Renmin Road, Kunming 650051, China; Department of Cardiovascular Surgery, Yan’an Affiliated Hospital of Kunming Medical University, No. 245 East Renmin Road, Kunming 650051, China; Institute of Cardiovascular Surgery of Yunnan Province, No. 245 East Renmin Road, Kunming 650051, China; Department of Cardiovascular Surgery, Yan’an Affiliated Hospital of Kunming Medical University, No. 245 East Renmin Road, Kunming 650051, China; Institute of Cardiovascular Surgery of Yunnan Province, No. 245 East Renmin Road, Kunming 650051, China; Department of Cardiovascular Surgery, Yan’an Affiliated Hospital of Kunming Medical University, No. 245 East Renmin Road, Kunming 650051, China; Institute of Cardiovascular Surgery of Yunnan Province, No. 245 East Renmin Road, Kunming 650051, China; Department of Cardiovascular Surgery, Yan’an Affiliated Hospital of Kunming Medical University, No. 245 East Renmin Road, Kunming 650051, China; Institute of Cardiovascular Surgery of Yunnan Province, No. 245 East Renmin Road, Kunming 650051, China

**Keywords:** Mitral stenosis, Heart failure, Left ventricular rupture, Nausea, Pulmonary hypertension, Mechanical valve dysfunction, Case report

## Abstract

**Background:**

Mitral valve surgery in patients with chronic severe heart failure carries a high perioperative risk. Life-threatening complications such as intraoperative left ventricular rupture, severe postoperative ventricular dysfunction, and subsequent mechanical valve thrombosis remain challenging and require urgent multidisciplinary management.

**Case summary:**

A 57-year-old woman with severe rheumatic mitral stenosis, atrial fibrillation, and New York Heart Association class III heart failure underwent mitral valve replacement, tricuspid annuloplasty, left atrial thrombectomy, and left atrial appendage closure. Intraoperative acute left ventricular rupture was successfully repaired urgently. Postoperatively, she developed severe left ventricular dysfunction (left ventricular ejection fraction [LVEF], 22%) requiring intra-aortic balloon pump support on postoperative day 1 (POD 1), followed by successful extubation (POD 11), stress-induced gastrointestinal bleeding (POD 13), device removal (POD 14), and prosthetic valve thrombosis (POD 20) managed by intensified anticoagulation. Ventricular and valve function improved markedly by POD 29 (LVEF 43%; effective orifice area, 1.7 cm^2^). At 3- and 5-month follow-ups, optimized medical therapy maintained stable cardiac function, and the patient remained asymptomatic.

**Discussion:**

This case highlights critical surgical and postoperative challenges associated with long-standing rheumatic mitral stenosis. Despite preserved preoperative LVEF, the patient developed profound haemodynamic instability requiring mechanical circulatory support. Additionally, the coexistence of gastrointestinal bleeding and prosthetic valve thrombosis required a carefully balanced and individualized anticoagulation strategy. This study emphasizes the importance of early recognition of risk factors for ventricular rupture, prompt surgical intervention, and multidisciplinary collaboration in managing complex cardiac cases. Furthermore, it underscores the role of conservative management with intensified anticoagulation when reoperation or thrombolysis is not feasible.

Learning points

- Severe chronic mitral stenosis predisposes patients to left ventricular (LV) atrophy and increases intraoperative ventricular rupture risk.
- Severe postoperative LV dysfunction may occur despite preserved preoperative LV ejection fraction, necessitating early mechanical circulatory support.
- Mechanical valve thrombosis may manifest as leaflet restriction without clearly visualized thrombus; intensified combined anticoagulation is an effective management strategy.
- Multidisciplinary collaboration among cardiac surgery, cardiology, intensive care, and gastroenterology is crucial for managing overlapping life-threatening complications.

## Introduction

Mitral valve surgery remains the primary treatment for severe symptomatic rheumatic mitral stenosis; however, it carries substantial perioperative risk in patients with long-standing disease, chronic heart failure, and adverse ventricular geometry. Intraoperative left ventricular (LV) rupture is among the most catastrophic complications in cardiac surgery, with an incidence of up to 1% and a mortality rate approaching 85%.^1^

Although surgical prevention and repair techniques for LV rupture have been widely reported, perioperative management of patients surviving acute rupture remains unexplored, particularly when complicated by refractory ventricular dysfunction, gastrointestinal bleeding, and prosthetic valve thrombosis. The coexistence of these life-threatening complications presents significant clinical challenges, especially regarding haemodynamic support and anticoagulation management.

Herein, we present a high-risk patient with chronic rheumatic mitral stenosis who developed intraoperative LV rupture, severe LV dysfunction, stress-induced gastrointestinal bleeding, and mechanical prosthetic valve thrombosis. This case highlights multidisciplinary critical care and emphasizes key management strategies for complex complications in high-risk mitral valve surgery.

## Summary figure

**Figure:**
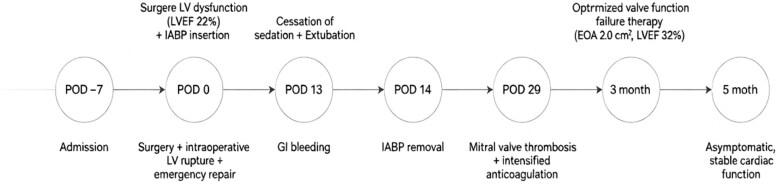
EOA, effective orifice area; GI, gastrointestinal; IABP, intra-aortic balloon pump; LVEF, left ventricular ejection fraction; POD, postoperative day.

## Case presentation

**Figure 1:**
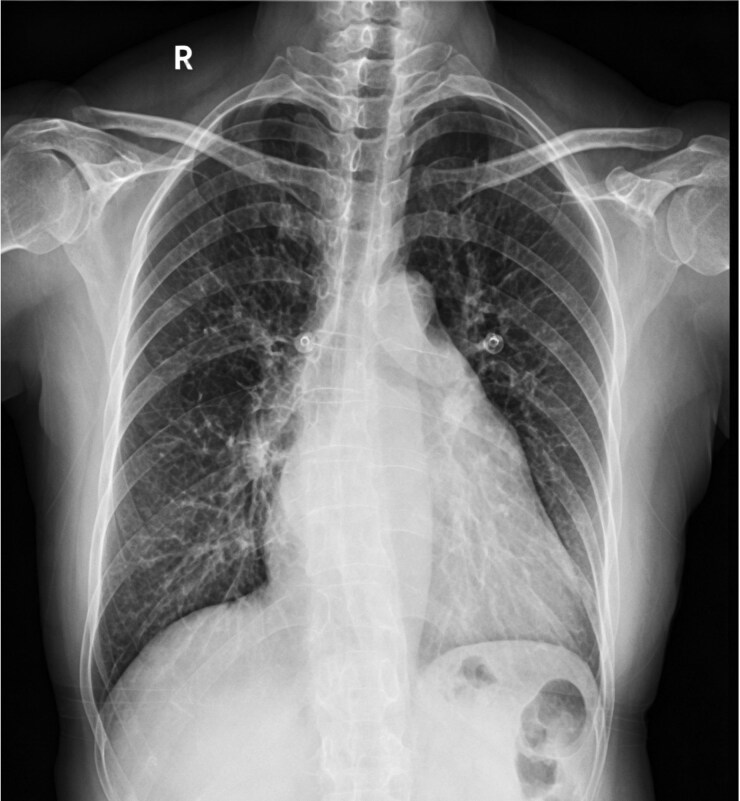
Preoperative chest radiographs.

A 54-year-old woman presented with a 10-day history of progressive exertional dyspnoea and bilateral lower-extremity oedema, abdominal distension, and nausea. She was admitted on 29 October 2025. She had no history of hypertension, diabetes mellitus, or other chronic diseases. Physical examination revealed an irregular heart rhythm of 89 beats/min and blood pressure of 120/80 mmHg. Her weight and height were 45 kg and 158 cm, respectively. Transthoracic echocardiography demonstrated severe rheumatic mitral stenosis with mild regurgitation, left atrial enlargement, pulmonary hypertension (approximately 46 mmHg), mild tricuspid regurgitation, preserved LV ejection fraction (LVEF) of 50%, and a LV end-diastolic diameter of 50 mm. Electrocardiography (ECG) confirmed atrial fibrillation (AF). Chest radiography demonstrated pulmonary venous congestion (*Figure 1*) and a double atrial shadow. She had New York Heart Association class III heart failure. Therefore, preoperative management included diuresis and electrolyte correction.

On 5 November 2025, the patient underwent mitral valve replacement, tricuspid annuloplasty, left atrial thrombectomy, and closure of the left atrial appendage orifice under cardiopulmonary bypass (CPB). Intraoperatively, a left atrial appendage thrombus (3.5 × 3 cm) was identified and excised. The mitral valve showed severe thickening and calcification with fusion of the chordae tendineae and papillary muscles. Only partial posterior leaflet and subvalvular structures were preserved, and a 27-mm ATS mechanical prosthetic valve was implanted. Tricuspid Kay annuloplasty was performed, and rapid left atrial backflow with central venous pressure >15 mmHg was observed.

**Figure 2:**
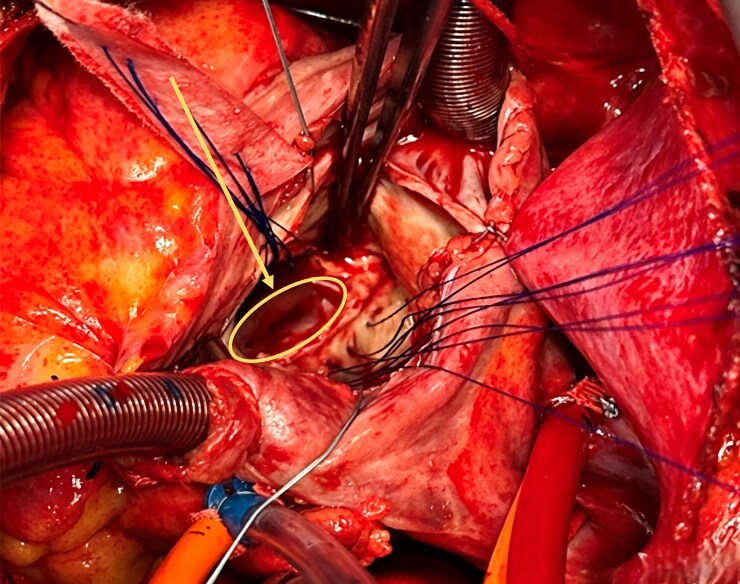
Left ventricular rupture.

After successful weaning from CPB, the chest was closed routinely; however, acute hypotension with increased pericardial and mediastinal drainage developed subsequently. Emergency resternotomy and reinstitution of CPB revealed LV rupture near the left atrial appendage, with an endocardial laceration located 1.5 cm from the P1 scallop (*Figure 2*). The mechanical valve was explanted, and the ventricular rupture was repaired using felt strips and bovine pericardial patches to reinforce epicardial and endocardial defects. A 27-mm ATS mechanical mitral valve was reimplanted. Total CPB and aortic cross-clamp times were 352 and 170 min, respectively. Intraoperative blood loss was 1400 mL.

On postoperative day 1 (POD 1), the patient developed severe haemodynamic instability. Bedside echocardiography demonstrated an LVEF of 22%. Thus, an intra-aortic balloon pump (IABP) was inserted for circulatory support, and broad-spectrum antibiotics were initiated.

Given a similar previous case in which recurrent LV rupture and death occurred after awakening following 5 days of sedation, prolonged sedation was maintained for 11 days until the patient regained full consciousness before extubation. On POD 13, the patient exhibited poor wound healing and intermittent melena, consistent with stress-induced gastrointestinal ulcer bleeding; gastroenterology consultation recommended nil per os status, proton pump inhibitors, somatostatin, and thrombin lavage. Warfarin was discontinued, and low-molecular-weight heparin (LMWH) was initiated for thromboprophylaxis. Haemodynamic status improved on POD 14, allowing IABP removal.

**Figure 3:**
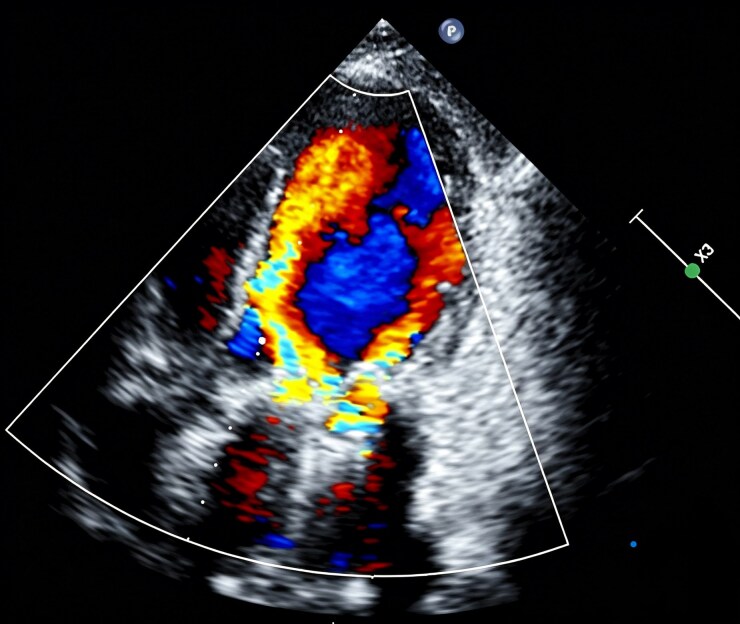
Ultrasound image of mechanical valve stenosis.

On POD 20, recurrent haemodynamic instability occurred. Echocardiography revealed restricted leaflet motion of the mitral mechanical prosthesis, with an orifice area of 0.8 cm^2^, suggesting prosthetic valve thrombosis (*Figure 3*). ECG demonstrated AF with abnormal Q waves in bipolar limb leads I (leads I) and augmented vector left arm lead (aVL). Multidisciplinary consultation concluded that interventional thrombolysis was not feasible. Intensified anticoagulation therapy was initiated with enoxaparin 4000 IU subcutaneously every 12 h, combined with warfarin. Gastrointestinal mucosal protection was continued. After 9 days, echocardiography revealed improved leaflet motion with a valve area of 1.7 cm^2^ and LVEF of 43%. The patient was discharged on oral warfarin therapy [target international normalized ratio (INR) of 2.0–2.5] plus daily 2000 IU LMWH. Only minor epistaxis was observed during follow-up.

**Figure 4:**
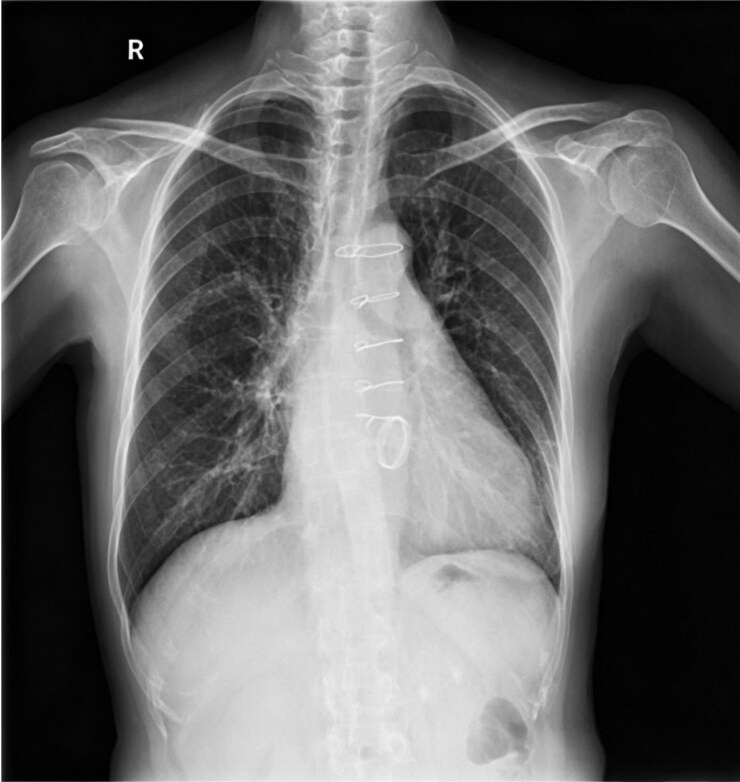
Chest radiographs at 3 months postoperatively.

At 3 months postoperatively, follow-up echocardiography demonstrated persistent restriction of the mitral mechanical prosthesis leaflet, with an orifice area of 2.0 cm^2^ and LVEF of 32%. AF persisted on ECG. Chest radiography revealed improvement in pulmonary congestion compared with baseline (*Figure 4*). Owing to declining cardiac function, guideline-directed medical therapy for heart failure was initiated. Warfarin plus indobufen was continued. At 5 months postoperatively, the patient remained asymptomatic. Long-term follow-up with serial echocardiography is planned.

## Discussion

**Figure 5:**
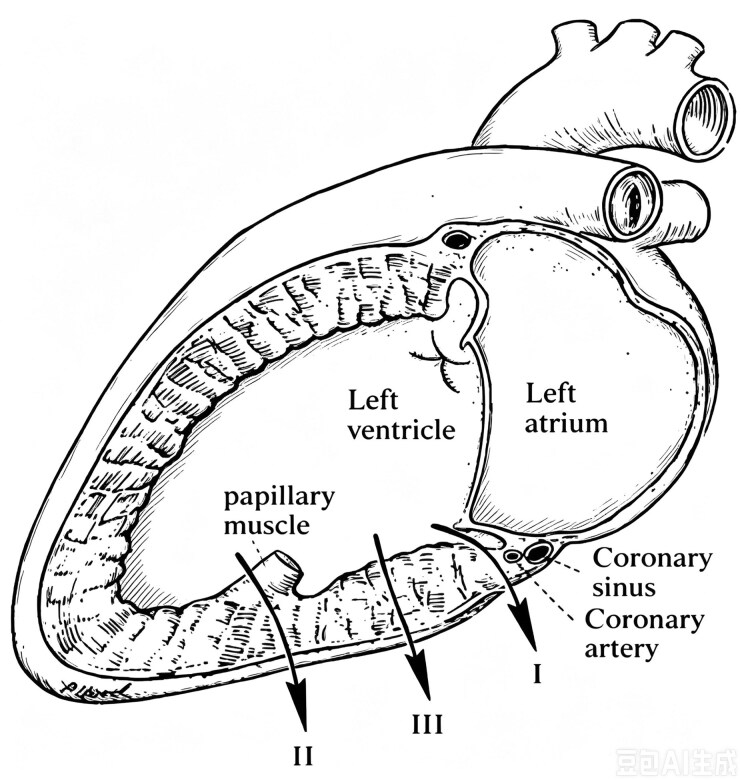
Miller classification.

Intraoperative LV rupture is a catastrophic complication commonly associated with chronic LV underfilling, myocardial fibrosis, a small LV cavity (<40 mm), malnutrition (body mass index <18.5 kg/m^2^), excessive resection of posterior leaflet tissue, implantation of an oversized prosthetic valve, central venous pressure of >15 mmHg, and excessive inotropic support (dopamine >10 μg/kg/min).^2^ According to the Miller classification (*Figure 5*),^3^ repair of type I rupture requires prosthetic valve explantation and double-layer repair using intracardiac pericardial patches while preserving the left circumflex coronary artery and coronary sinus. Epicardial reinforcement with felt strips in a sandwich configuration is recommended. Types II and III are repaired using full-thickness continuous sutures with felt-reinforced nontraumatic needles and an interposed biological patch. All repairs should be performed under CPB.^4–6^

Although previous studies have emphasized preventive measures and surgical repair techniques for LV rupture, postoperative management and treatment of related complications are equally essential for survival. This case demonstrates an extremely high-risk perioperative course in a patient with long-standing severe rheumatic mitral stenosis.

The patient exhibited advanced valvular and subvalvular disease, and only partial preservation of the posterior leaflet was possible during surgery. Additionally, rapid left atrial backflow and central venous pressure of >15 mmHg were identified as major contributors for acute LV rupture, which was successfully managed with emergency CPB and combined endocardial–epicardial repair.

Severe postoperative LV dysfunction developed, with an LVEF of 22%. Early IABP support stabilized haemodynamics. Although adequate sedation and strict bed rest may reduce the risk of rerupture, they may also increase the risk to complications such as pneumonia and thromboembolism.

Stress-induced gastrointestinal ulcer bleeding further complicated anticoagulation management, requiring a careful balance between thromboprophylaxis and haemostasis.

Lifelong anticoagulation is required after mechanical valve replacement. The target INR is 1.8–2.2 for mitral valve positions and 2.5–3.0 for high-risk patients. In patients at high risk of bleeding, antiplatelet agents may partially replace anticoagulant therapy, whereas patients with AF require enhanced anticoagulation. Suspected thrombosis should be confirmed by transoesophageal echocardiography, with thrombolysis or surgical intervention implemented accordingly.^7,8^ However, mechanical valve leaflet restriction and thrombosis occurred. Multidisciplinary evaluation revealed that reoperative thrombectomy carried a high risk; therefore, as thrombolysis was not feasible, a conservative approach with intensified dual anticoagulation was adopted.

This case underscores the importance of multidisciplinary critical care, prompt surgical intervention, mechanical circulatory support, and individualized anticoagulation strategies in managing complex rheumatic valve surgery.

### Patient perspective

The patient experienced progressive shortness of breath and leg swelling before surgery. She subsequently underwent surgical intervention with careful multidisciplinary management. Despite a complex perioperative course, she gradually recovered and remained asymptomatic at the 5-month follow-up. She expressed satisfaction with the treatment and provided informed consent for publication of this case report.

## Data Availability

The data underlying this article are available within the article.

## Lead author biography

Maodong yang graduated from Kunming Medical University and currently works in the Department of Cardiovascular Surgery, Yan'an Affiliated Hospital of Kunming Medical University, Kunming, China

## Notes

**Consent:** Written informed consent was obtained from the patient for publication of this case report and the accompanying images.
